# Work-related musculoskeletal complaints and ergonomic risk factors among Egyptian anesthesiologists: a cross-sectional study

**DOI:** 10.1186/s12889-024-17757-x

**Published:** 2024-01-23

**Authors:** Ahmed Mahmoud Fouad, Ayman Ekram Fahim, Ahmed Abdelmohsen Bedewy, Aiman Al-Touny, Shimaa A. Al-Touny

**Affiliations:** 1 Department of Public Health, Occupational & Environmental Medicine, Faculty of Medicine, Suez Canal University, Ismailia, Egypt; 2 Department of Anesthesiology & Critical Care, Faculty of Medicine, Helwan University, Cairo, Egypt; 3 Department of Anesthesiology & Critical Care, Faculty of Medicine, Suez Canal University, Ismailia, Egypt

**Keywords:** Musculoskeletal disorders, Anesthesiologists, Ergonomic, Egypt

## Abstract

**Background:**

Anesthesiologists are vulnerable to work-related musculoskeletal disorders (WMSDs) due to sustained repetitive movements and awkward postures. This study aimed to assess the prevalence of WMSDs among anesthesiologists and to evaluate its association with ergonomic risk factors.

**Methods:**

A convenience sample of 380 Egyptian anesthesiologists were invited to participate in this cross-sectional study through an electronic questionnaire. Data were collected from May to August 2022 and involved questions about participants’ demographic, health, and work-related characteristics; the ergonomic risks and perceived hazards; and the musculoskeletal complaints during the past 12 months and 7 days - using Nordic Musculoskeletal Questionnaire (NMQ). Descriptive, bivariate, and multivariate statistical analyses were used to estimate the prevalence of MSD and identify its determinants in the studied sample.

**Results:**

A total of 215 anesthesiologists were included in this study, with a 56.8% response rate, 66% males with an average age of 38 (± 0.7) years. 21% were resident physicians, 47% were specialists, and 32% were consultants. The 12-month prevalence of MSD among anesthesiologists was 71.6% (95% CI: 65.6– 77.7%). Multivariate analysis showed that the main determinants of MSD among the studied sample were age of 45-years and older (OR: 3.22, 95% CI: 1.21–8.52, *p* = 0.018), regular physical exercise (OR: 0.25, 95% CI: 0.10–0.65, *p* = 0.005), insufficient rest time between procedures (OR: 2.25, 95% CI: 1.15–4.41, *p* = 0.018), and three or more awkward postures of the trunk (OR: 3.55, 95% CI: 1.43–8.82, *p* = 0.006).

**Conclusions:**

The study highlights a high prevalence of WMSDs among Egyptian anesthesiologists, linked to advancing age, lack of regular exercise, insufficient rest between procedures, and frequent awkward postures. Addressing these ergonomic risk factors through targeted workplace interventions is crucial for promoting the overall well-being of anesthesiologists and ensuring the provision of safe anesthesia services.

## Background

Musculoskeletal disorders (MSDs) comprise injuries or disorders of the muscles, nerves, tendons, joints, cartilage, or spinal discs caused by sudden or sustained exposure to repetitive motion, force, vibration, and awkward positions [1].

Work-related musculoskeletal disorders (WMSDs) are prevalent among workers worldwide accounting for 42-58% of all work-related diseases [2]. The prevalence of WMSDs ranges from 35 to 47% in America, 54–60% in Europe, 79– 88% in Asia, and 44– 94% in Africa, with low-middle–income countries (LMIC) showing the highest prevalence [3–5]. WMSDs represent the most common causes of chronic disability, health-related absences, reduced work productivity, and compromised quality of life [6]. In 2020, 22.0% of global years lived with disability (YLDs) resulting from MSDs were attributable to exposure to occupational ergonomic factors, representing a total of 194 YLDs per 100 000 population. Furthermore, WMSDs is one of the major contributing factors to the economic burden, with an estimated total cost of lost productivity, due to sick leave, as high as 2% of gross domestic product (GDP) in the European Union countries [7, 8].

Anesthesiologists are vulnerable to WMSDs due to their excessive exposure to ergonomic risk factors and physical demands, such as repetitive tasks and awkward postures for prolonged periods [9, 10]. These factors, along with improper body mechanics, inadequate workspace design, and heavy lifting, contribute to the development of MSDs [9, 11]. Besides, psychological, and organizational factors, such as high job stress, long working hours, and irregular work schedules, can also influence the occurrence of WMSDs among anesthesiologists [12].

The health and wellbeing of healthcare workers are crucial for healthcare systems’ performance [13]. WMSDs can negatively impact their well-being, patient safety, and the overall quality of care. WMSDs can lead to job dissatisfaction, burnout, decreased productivity, and potentially hinder their ability to provide optimal patient care [14]. Earlier studies showed a high prevalence of WMSDs among anesthesiologists; ranged from 34 to 98.4% [15–17]. The lower back, neck, and shoulders were the most frequently affected body regions [15, 16]. Limited studies in Arab countries have estimated the 12-months prevalence of WMSDs among anesthesiologists as high as 82.4% and 100% in Saudi Arabia and Egypt, respectively [18, 19]. However, information on ergonomic risk factors among anesthesiologists is still lacking, particularly in these countries. A recent study was conducted in Egypt by Albayadi et al. [18] as a single institution experience, in a university hospital, focusing on the prevalence of WMSDs among anesthesiologists but did not address the ergonomic risk factors. Another study by Tolu and Basaran [16] addressed the prevalence and ergonomic risk factors of WMSDs among anesthesiologists in the Turkish context. However, cultural and health system differences across countries may affect the burden of the WMSDs problem and the mitigation activities.

Therefore, the purpose of our study was to determine the prevalence and the work-related risk factors, with special emphasis on ergonomic factors, contributing to WMSDs among anesthesiologists in Egypt. Addressing these factors in the Egyptian context allows for comparisons with other countries and helps healthcare organizations promoting a healthier work environment, reducing absenteeism due to WMSDs, and enhancing overall performance. Besides, it is crucial for ensuring the provision of safe anesthesia care.

## Methods

This cross-sectional study was conducted from May to August 2022. A total of 380 Egyptian anesthesiologists were electronically accessible through social media professional groups. An invitation link to the electronic questionnaire, offered in English, was sent to all accessible and eligible anesthesiologists. Eligible participants included anesthesiologists who had at least one year of working experience and did not have any pre-existing autoimmune diseases that affect their joints (e.g., rheumatoid arthritis, Psoriatic arthritis, ankylosing spondylitis, and systemic lupus erythematosus). A total of 215 anesthesiologists responded and were eligible to participate in this study, with a 56.8% response rate. A sample size calculation using OpenEpi online sample size calculator (available at: https://www.openepi.com/SampleSize/SSPropor.htm) emphasized that a sample of 215 participants was large enough to detect a prevalence of WMSDs among anesthesiologists of at least 34% [15]– at 95% level of confidence, 5% absolute precision level, 380 population size, and 15% non-response rate.

The questionnaire included questions about participants’ demographic (age, gender, marital status), lifestyle (smoking and physical exercise), and health characteristics (handedness, chronic illnesses, perceived general health status). Work-related characteristics included work duration, professional level, specialties of practice, workload (e.g., working hours per week, night shifts, number of procedures per week, average duration of procedures, rest-time between procedures, and assistance by other personnel during procedures). The number of working hours per week was classified as: less than 35 h (part-time), 35–40 (standard), 41–54 (long working hours), and 55 or more (extra-long working hours) [20]. Participants were asked about how they perceived the MSDs risks (low, moderate, high) given the quality of the ergonomic conditions at the operating room (i.e., if they satisfying/ responding to their work tasks needs/ movements/ maneuvers). Additionally, participants were asked to identify the postures that best describe their neck and upper extremities, trunk, and lower extremities during work. Then, the number of awkward postures of each body region during work was calculated as single, two, and three or more.

WMSDs were measured by the English version of Standardized Nordic Musculoskeletal Questionnaire (NMQ) [21]. It is a validated, easy to use, short instrument to assess self-reported work-related musculoskeletal complaints (ache, pain, discomfort, or numbness) in different body regions (neck, shoulder, elbows, wrists/hands, upper back, lower back, hips, knees, and ankles/feet) during the past 12 months and 7 days. Additionally, the NMQ assesses the severity of complaints by asking about the need for a physician visit, physiotherapy, medical treatment or getting sick leave.

All statistical analyses were performed using Statistical Package for the Social Sciences (SPSS version 28.0; IBM Corporation, Armonk, NY, USA). Categorical variables were presented as frequencies and percentages (%) while continuous variables were summarized as mean and standard deviation (SD). The prevalence of WMSDs was presented as a proportion and 95% confidence interval. Association between categorical variables were tested for statistical analysis by Chi-square test or Fisher’s exact test (if > 20% of expected values were less than 5). Logistic regression analysis was used to test for adjusted associations between the WMSDs (Dependent variable) study variables that yielded a statistical significance of 0.20 or less on bivariate analyses. A *p*-value of less than 0.05 was considered statistically significant.

## Results

A total of 215 anesthesiologists responded and were included in this study, with a 56.8% response rate. They aged 25–65 years with a mean of 38.0 (± 9.7) years, and females comprised 34.0% of the sample. Further demographic, lifestyle and health characteristics are presented in Table 1.

**Table 1:** Sociodemographic and work-related characteristics of the studies sample (*N* = 215)

Characteristics		Frequency (%)
Age (years)	Mean (SD), range. < 30 30–39 40–49 50–59 60 +	38.0 (9.7), 22–75 28 (13.0%) 111 (51.6%) 53 (24.7%) 13 (6.0%) 10 (4.7%)
Gender	Male Female	142 (66.0%) 73 (34.0%)
Marital status	Single Married Divorced/ Widow/ Separated	48 (22.3%) 161 (74.9%) 6 (2.8%)
Smoking	Non-smoker Current smoker Ex-smoker	158 (73.5%) 30 (14.0%) 27 (12.7%)
Physical exercise	None Regular Irregular	140 (65.1%) 27 (12.6%) 48 (22.3%)
Handedness	Right Left	199 (92.6%) 16 (7.4%)
Chronic illnesses	No Yes	166 (77.2%) 49 (22.8%)
Perceived general health status:	Low Average High	9 (4.2%) 126 (58.6%) 80 (37.2%)
Years working as anesthesiologist:	Mean (SD) < 5 5–14 15–24 25 +	12.2 (9.4) 40 (18.6%) 104 (48.4%) 49 (22.8%) 22 (10.2%)
Current professional level:	Resident Specialist Consultant	46 (21.4%) 100 (46.5%) 69 (32.1%)
Main specialties of practice:	Obstetrics and Gynecology Pediatric surgery Orthopedics surgery Cardiothoracic surgery Neurosurgery ICU/ Pain management Others	144 (67.0%) 98 (45.6%) 131 (60.9%) 41 (19.1%) 91 (42.3%) 77 (35.8%) 56 (26.0%)
Number of working hours per week:	< 35 (Part-time) 35–40 (Standard hours) 41–54 (Long hours) 55 + (Extra Long hours)	23 (10.7%) 22 (10.2%) 54 (25.1%) 116 (54.0%)
Number of night shifts per month:	None 1–4 5 +	53 (24.7%) 71 (33.0%) 91 (42.3%)
Number of procedures per week:	< 5 5–15 16–24 25 +	6 (2.8%) 75 (34.9%) 50 (23.3%) 84 (39.1%)
Average duration of procedures:	< 1 h 1–3 h > 3 h	14 (6.5%) 166 (77.2%) 35 (16.3%)
Rest time between procedures:	Not enough Enough	143 (66.5%) 72 (33.5%)
Having assistant personnel during procedures?	No Yes	93 (43.3%) 122 (56.7%)

21% were resident physicians, 47% were specialists, and 32% were consultants, with a mean work duration of 12.2 (± 9.4) years. Obstetrics and Gynecology, and Orthopedics were the main areas of practice in 67.0% and 60.9% of the sample, respectively. About 80% were experiencing extended work hours (> 40 h per week), with 54.0% working for 55 h or more per week. Night shift was reported by 75.0% of the study participants, with 42.3% had at least five nightshifts per month. Two-thirds of the participants averaged more than 15 procedures per week, with 77.2% lasting 1–3 h, 66.5% experiencing insufficient rest, and 43.3% getting no assistance (Table 1).

All participants have reported WMSDs in the past 7 days, with lower back and knees being the most frequently affected body regions (54.4% and 49.8%, respectively). However, the12-month prevalence of WMSDs among anesthesiologists of this study is estimated as 71.6% (95% CI: 65.6– 77.7%), with lower back, neck, and shoulders being the most affected body regions (51.2%, 28.4%, and 26.5%, respectively). Over two-thirds of the participants (39.1%) reported WMSDs in two or more body regions in the past 12 months. Over half of the participants (57.1%) with a 12-month history of WMSDs, the complaint was severe enough to seek medical care (i.e., clinic visit, medications, or physiotherapy) or get a sick leave (Table 2).

**Table 2:** Work-related musculoskeletal disorders (WMSDs) and ergonomic risk factors (*N* = 215)

Characteristics		Frequency (%)
**WMSDs in the past 7 days**		
None Low Back Knees Neck Shoulder Wrist/ Hand Elbows		0 117 (54.4%) 107 (49.8%) 79 (36.7%) 69 (32.1%) 26 (12.1%) 3 (1.4%)
**WMSDs in the past 12 months**		
None Low Back Neck Shoulder Knees Wrist/ Hand Elbows		61 (28.4%) 110 (51.2%) 61 (28.4%) 57 (26.5%) 43 (20.0%) 25 (11.6%) 5 (2.3%)
**Number of musculoskeletal regions affected in the past 12 months**:		
None One region Two regions Three or more regions		61 (28.4%) 70 (32.6%) 40 (18.6%) 44 (20.5%)
**Visited a Physician, took medications or physiotherapy or got sick leave for** **WMSDs in the past 12 months** (***n*** = **154**):		
No Yes		66 (42.9%) 88 (57.1%)
**Perceived WMSDs risks at the operating room**		
Low Moderate High Cannot determine		40 (18.6%) 121 (56.3%) 47 (21.9%) 7 (3.3%)
**Postures during work** ***Trunk***		
Standing Sitting Turn round frequently Keep the same posture for long time Keep bending for long time Bend and turn at the same time frequently Heavy lifting of instruments (≥ 2 kg)		167 (77.7%) 64 (29.8%) 61 (28.4%) 46 (21.4%) 42 (19.5%) 39 (18.1%) 13 (6.0%)
***Neck & Upper Extremities***		
Turn the head frequently Keep the head low for long time Intermittently flexed neck and elevated arms Repetitive wrist flexion and extension Repetitive and forceful gripping Keep the neck twisted for long time Twist your arm frequently Arm placed on edges of angular objects Keep the wrist twisted for a long time Have support device in your forearm		91 (42.3%) 90 (41.9%) 60 (27.9%) 51 (23.7%) 36 (16.7%) 28 (13.0%) 21 (9.8%) 19 (8.8%) 12 (5.6%) 7 (3.3%)
***Lower Extremities***		
Standing on the table Sitting Standing on the tiptoe		145 (67.4%) 86 (40.0%) 24 (11.2%)
**Number Awkward Postures during work**		
***Trunk***	Single Two Three or more	82 (38.1%) 71 (33.3%) 62 (28.8%)
***Neck & Upper Extremities***	Single Two Three or more	105 (48.8%) 55 (25.6%) 55 (25.6%)
***Lower Extremities***	Single Two Three or more	177 (82.3%) 36 (16.7%) 2 (0.9%)

Regarding the ergonomic risk factors, 21.9% of participants described the operating rooms as not fitting the needs of their work tasks, perceiving the risk for WMSDs as high. Different postures of each body region were reported by the study participants and presented in Table 2. Over one-fourth of participants reported three or more awkward postures during work at their trunk (28.8%), neck and upper extremities (25.6%), while only two participants (0.9%) reported three or more awkward postures at their lower extremities (Table 2).

Bivariate analyses of the associations between WMSDs and different demographic, lifestyle, health, and work-related characteristics, and ergonomic factors are presented in Table 3. The WMSDs were significantly associated with participants’ perceived general health status (*p* = 0.026), adequacy of rest time between procedures (*p* = 0.015), and the number of trunk’s awkward postures (*p* = 0.006). In Table 4, multivariate analysis with a logistic regression model showed that the main determinants of WMSDs among the studied sample of anesthesiologists were: age of 45-years and older (OR: 3.22, 95% CI: 1.22–8.52, *p* = 0.018), regular physical exercise (OR: 0.25, 95% CI: 0.10–0.65, *p* = 0.005), insufficient rest time between procedures (OR: 2.25, 95% CI: 1.15–4.41, *p* = 0.018), and three awkward postures of the trunk (OR: 3.55, 95% CI: 1.43–8.82, *p* = 0.006) with a significant dose-response relationship (*p* for trend = 0.01).

**Table 3:** Associations between WMSDs and all study variables (*N* = 215)

Characteristics		WMSDs, n (%)		*p*-value
		**No** (*n* = 61)	**Yes** (*n* = 154)	
Age (years)	< 45 ≥ 45	53 (86.9%) 8 (13.1%)	122 (79.2%) 32 (20.8%)	0.193
Gender	Male Female	46 (75.4%) 15 (24.6%)	96 (62.3%) 58 (37.7%)	0.068
Marital status	Single Married Divorced/ Widow/ Separated	10 (16.4%) 48 (78.7%) 3 (4.9%)	38 (24.7%) 113 (73.4%) 3 (1.9%)	0.216 ^F^
Smoking	Non-smoker Current smoker Ex-smoker	45 (73.8%) 8 (13.1%) 8 (13.1%)	113 (73.4%) 22 (14.3%) 19 (12.3%)	0.968
Physical exercise	None Regular Irregular	36 (59.0%) 13 (21.3%) 12 (19.7%)	104 (67.5%) 14 (9.1%) 36 (23.4%)	0.051
Handedness	Right Left	54 (88.5%) 7 (11.5%)	145 (94.2%) 9 (5.8%)	0.161 ^F^
Chronic illnesses	No Yes	50 (82.0%) 11 (18.0%)	116 (75.3%) 38 (24.7%)	0.295
Perceived general health status	Low Average High	3 (4.9%) 27 (44.3%) 31 (50.8%)	6 (3.9%) 99 (64.3%) 49 (31.8%)	0.026*
Years working as anesthesiologist	< 5 5–14 15–24 25 +	11 (18.0%) 34 (55.7%) 9 (14.8%) 7 (11.5%)	29 (18.8%) 70 (45.5%) 40 (26.0%) 15 (9.7%)	0.319
Current professional level	Resident Assistant Lecturer (Specialist) Lecturer + (Consultant)	12 (19.7%) 30 (49.2%) 19 (31.1%)	34 (22.1%) 70 (45.5%) 50 (32.5%)	0.873
Main specialties of practice	Obstetrics and Gynecology Pediatric surgery Orthopedics surgery Cardiothoracic surgery Neurosurgery ICU/ Pain management Others	36 (59.0%) 25 (41.0%) 31 (50.8%) 13 (21.3%) 21 (34.4%) 21 (34.4%) 12 (19.7%)	108 (70.1%) 73 (47.4%) 100 (64.9%) 28 (18.2%) 70 (45.5%) 56 (36.4%) 44 (28.6%)	0.118 0.394 0.056 0.598 0.140 0.789 0.180
Working hours per week	< 35 (Part-time) 35–40 (Standard hours) 41–54 (Long hours) 55 + (Extra Long hours)	5 (8.2%) 8 (13.1%) 18 (29.5%) 30 (49.2%)	18 (11.7%) 14 (9.1%) 36 (23.4%) 86 (55.8%)	0.531
Night shifts per month	None 1–4 5 +	12 (19.7%) 24 (39.3%) 25 (41.0%)	41 (26.6%) 47 (30.5%) 66 (42.9%)	0.382
Number of procedures per week	< 5 5–15 16–24 25 +	3 (4.9%) 21 (34.4%) 12 (19.7%) 25 (41.0%)	3 (1.9%) 54 (35.1%) 38 (24.7%) 59 (38.3%)	0.587 ^F^
Average duration of procedures	< 1 h 1–3 h > 3 h	3 (4.9%) 45 (73.8%) 13 (21.3%)	11 (7.1%) 121 (78.6%) 22 (14.3%)	0.409
Rest time between procedures	Not enough Enough	33 (54.1%) 28 (45.9%)	110 (71.4%) 44 (28.6%)	0.015*
Having assistant personnel during procedures?	No Yes	26 (42.6%) 35 (57.4%)	67 (43.5%) 87 (56.5%)	0.906
Perceived ergonomic hazards at the operating room	None Moderate High Cannot determine	13 (21.3%) 37 (60.7%) 10 (16.4%) 1 (1.6%)	27 (17.5%) 84 (54.5%) 37 (24.0%) 6 (3.9%)	0.480
Number Awkward Postures of the Trunk	Single Two Three or more	28 (45.9%) 25 (41.0%) 8 (13.1%)	54 (35.1%) 46 (29.9%) 54 (35.1%)	0.006*
Number Awkward Postures of the Neck and Upper Extremities	Single Two Three or more	35 (57.4%) 13 (21.3%) 13 (21.3%)	70 (45.5%) 42 (27.3%) 42 (27.3%)	0.289
Number Awkward Postures of the Lower Extremities	Single Two Three or more	48 (78.7%) 13 (21.3%) 0	129 (83.8%) 23 (14.9%) 2 (1.3%)	0.366 ^F^

*. Statistically significant *p*-value at *p* < 0.05. Default test in all associations was Chi-square test. ^F^. Fisher’s Exact test

**Table 4:** Stepwise backward binary logistic regression model of the predictors of WMSDs among anesthesiologists (*N* = 215)

	B	S.E.	Wald	df	*p*-value	OR	95% CI
**Age ≥ 45 years**	1.17	0.50	5.57	1	0.018*	**3.22**	**1.22– 8.52**
**No physical exercise** (Ref)			8.69	2	0.013*		
**Regular physical exercise**	-1.38	0.49	7.95	1	0.005*	**0.25**	**0.10–0.66**
**Irregular physical exercise**	0.11	0.41	0.08	1	0.781	1.12	0.50–2.51
**Insufficient rest time between procedures**	0.81	0.41	5.61	1	0.018*	**2.25**	**1.15–4.41**
**Single Trunk Awkward Posture** (Ref)			8.47	2	0.014*		
**Two Trunk Awkward Posture**	0.02	0.36	0.04	1	0.950	1.02	0.50–2.07
**Three Trunk Awkward Posture**	1.27	0.47	7.41	1	0.006*	**3.55**	**1.43–8.82**
***Constant***	0.10	0.35	0.08	1	0.782	1.10	

**Variables entered on step 1**: age ≥ 45, gender, physical exercise, handedness, perceived general health status, obstetrics & gynecology related procedures, orthopedics related procedures, neuro procedures, other anesthetic specialties, enough resting time between procedures, and number of trunk’s awkward postures

Correct Class by the final step of the model %: 73.5%

Model fit (last step): Chi-square = 27.52, df, 6, *p* < 0.001

Nagelkerke R-square (last step): = 0.172

Hosmer & Lemshow test (last step): Chi-square = 1.24, df = 7, *p* = 0.990

*. Statistically significant *p*-value at *p* < 0.05

## Discussion

Anesthesiologists are particularly susceptible to WMSDs which is considered an impending epidemic given the imminent workforce shortage and increasing economic burden. However, the reported prevalence and risk factors differ between studies [16, 22]. Our study findings suggest a high prevalence of WMDs amongst Egyptian anesthesiologists (71.6%), with 51.2% reporting lower back pain in the past 12 months, 57.1% was severe enough to seek medical care or sick leave, and 100% having MSDs complaint at least once a week. The number of awkward postures during work was the strongest predictor of WMSDs, where the odds of MSDs complaints in the past 12 months was about four times higher among anesthesiologists experiencing three or more awkward postures compared to those experiencing a single awkward posture. Other predictors of WMSDs in our sample were increasing age, lack of regular physical exercise, and insufficient rest between procedures.

Earlier studies showed a high variability in the estimated 12-month prevalence of WMSDs among anesthesiologists. In our study, the estimated 12-month prevalence of WMSDs among anesthesiologists is 71.6%, which is comparable to the reported worldwide values that ranged from 34 to 98.4% [15, 16, 22]. Among the Arab countries, our finding is lower than the estimated prevalence of low back pain among anesthesiologists (82.4%) in Saudi Arabia [19]. Albayadi et al., a recent study in Egypt, reported that all anesthesiologists had at least one MSDs complaint in the past 12 months [18]. Though, the actual incidence of WMSDs may be significantly higher due to underreporting [23]. The underreporting of WMSDs among healthcare workers is multifactorial. It involves intentional concealment due to the fear of job loss, the avoidance of filing workers’ compensation claims, and the inadequate benefits provided by workers’ compensation, which creates an economic incentive for underreporting work-related injuries. Instead, healthcare workers may opt for seeking care and wage replacement through health insurance and paid personal time. Beyond intentional concealment, other reasons include the failure to correctly diagnose or recognize a disorder as work-related, a physician’s preference to self-manage the condition or rely on over-the-counter medications, employers suppressing reporting through administrative systems, and the compilation of inaccurate injury logs to avoid safety inspections [24].

In our study, the lower back was the most frequently reported body region for WMSDs among anesthesiologists in the past 12 months. This finding is comparable to a recent Turkish study by Tolu and Basaran [16], and a systematic review by Tavakkol et al. [25] The later reported that low back was the most prevalent MSDs among operating room personnel with a pooled global estimate of 61.5%, and individual studies ranging from 5.5 to 84.0%. Moreover, WMSDs of the lower back was the most frequent complaint among our study participants in the past 7 days, which is in line with findings from Tolu and Basaran [16]. Increased prevalence of lower back complaints might be attributed to the extreme rotational movements of the spine and overreaching for objects in key anesthetic procedures such as spinal anesthesia [23]. Though, Leifer et al. [15] reported that the nature of many anesthesia-related tasks poses greater risk of poor posture for the neck and upper limbs.

WMSDs are the leading cause of disability, absenteeism, and reduced productivity among healthcare workers. A 2018 meta-analysis by Epstein et al. [22] reported that 12% of physicians required a leave of absence, work restriction or modification, or ended up early retirement. However, our study showed that over half of participants required sick leave or medical care due to their WMSDs. This finding is comparable to other earlier studies with a percentage ranged from 43.9 − 65.9% [16, 18].

The etiology of WMSDs is multifactorial and has been best explained by the biopsychosocial model of health, founded by Waddell in 1987 [13, 26, 27], WMSDs are influenced by individual factors such as sex, comorbidities, job design, workplace environment, overwork, fatigue, and stress levels of employees [12, 27]. In addition, anesthesiologists are subject to ‘double burden’ which mandates balancing simultaneously the demands of multiple paid work with unpaid domestic-family work [12]. Our study findings suggest that anesthesiologists are facing challenging work schedules and workloads, with 79.1% working for extended hours (≥ 40 h per week), predominantly night shifts, and about two thirds were overloaded with numerous lengthy procedures per week with insufficient rest. However, the rest time between procedures was the only significant predictor for WMSDs where insufficient rest time is associated with 2.25 times greater odds of WMSDs compared to sufficient rest time. These findings are in line with Tolu and Basaran study [16] which reported 2.62 greater odds of WMSDs with insufficient rest time. Lack of association with gender, extended work hours, night shifts, and excess lengthy procedures contradicts many earlier studies [16, 28–30].

Anesthesiologists have a high risk for WMSDs due to sustained repetitive or forceful movements, static and awkward postures, and challenges with instrument design [10, 15, 17, 31]. The present study identified prolonged standing, repetitive head movement, head low position or tilting as the most frequent awkward postures among anesthesiologists. Besides, the increasing number of experienced awkward postures significantly predicts WMSDs where having at least three awkward postures is associated with 3.5 times greater odds of WMSDs compared to a single awkward posture. This observation is consistent with earlier studies demonstrating the ergonomic risk factors for WMSDs [16, 28, 32].

Although the literature suggest that young less experienced anesthesiologists are more likely to adopt awkward postures during procedures, [15] our study findings showed that older (≥ 45 years) anesthesiologists had significantly 3.22 times greater likelihood for WMSDs compared to younger participants. This finding can be attributed to the age-related changes in individual biomechanical tolerance to strength and loads, and the cumulative nature of ergonomic-related MSDs [12, 33]. Besides, regular exercise is recommended to alleviate fatigue resulting from working for extended hours and increased workloads among anesthesiologists [34]. Our findings support this recommendation since regular physical exercise among study participants was significantly associated with a 75% reduction in the odds of WMSDs compared to lack of exercise.

Several limitations should be considered while interpreting our study findings. Selection and misclassification bias are the major limitations of this study. Although the response rate of our study was acceptable, the convenience sampling using electronically accessible candidates increases the likelihood of selection bias. The use of a self-report instrument may increase the likelihood of participants responding if they had symptoms. However, the NMQ is a validated tool for WMSDs screening. Furthermore, the double-burden [12] is common in developing countries which may increase the liability for misclassification bias. The cross-sectional design of this study hinders causal inference due to the absence of information on the temporal relationship between the outcome and exposure. Finally, the study did not assess psychosocial factors as a significant factor contributing to WMSD according to the biopsychosocial model.

## Conclusions

This study suggests that anesthesiologists in Egypt are at substantial occupational health risk due to the high prevalence of WMSDs, particularly with increasing age, lack of regular exercise, lack of enough rest time between procedures, and increasing number of awkward postures at work. Addressing these factors, particularly ergonomic risk factors, is crucial for developing interventions and preventive strategies to maintain the well-being of anesthesiologists and ensuring the provision of safe and quality anesthesia services. Such interventions include, but are not limited to, the implementation of targeted ergonomic training programs, modification of workstations, promotion of regular breaks, investment in ergonomic equipment, introduction of wellness programs incorporating physical activity, ensuring easy access to occupational health services, improving reporting mechanisms, and monitoring ergonomic risk factors.

## Data Availability

The datasets analyzed during the current study are available from the corresponding author on reasonable request.

## Abbreviations

MSDs: Musculoskeletal Disorders
WMSDs: Work-related Musculoskeletal Disorders
NMQ: Nordic Musculoskeletal Questionnaire
